# Complete Genome Sequence of *Nissabacter* sp. Strain SGAir0207, Isolated from an Air Sample Collected in Singapore

**DOI:** 10.1128/MRA.00559-19

**Published:** 2019-08-08

**Authors:** Nicolas E. Gaultier, Ana Carolina M. Junqueira, Akira Uchida, Rikky W. Purbojati, James N. I. Houghton, Caroline Chénard, Anthony Wong, Sandra Kolundžija, Megan E. Clare, Kavita K. Kushwaha, Alexander Putra, Carmon Kee, Balakrishnan N. V. Premkrishnan, Cassie E. Heinle, Serene B. Y. Lim, Vineeth Kodengil Vettath, Daniela I. Drautz-Moses, Stephan C. Schuster

**Affiliations:** a Singapore Centre for Environmental Life Sciences Engineering, Nanyang Technological University, Singapore; b Departamento de Genética, Instituto de Biologia, Universidade Federal do Rio de Janeiro, Rio de Janeiro, Brazil; University of Maryland School of Medicine

## Abstract

Nissabacter sp. strain SGAir0207 was isolated from a tropical air sample collected in Singapore. Its genome was assembled using a hybrid approach with long and short reads, resulting in one chromosome of 3.9 Mb and 7 plasmids. The complete genome consists of 4,403 protein-coding, 84 tRNA, and 22 rRNA genes.

## ANNOUNCEMENT

Nissabacter spp. are rod-shaped, facultatively anaerobic, Gram-negative bacteria (1) and members of the *Enterobacteriaceae* family. The genus Nissabacter contains only one species, Nissabacter archeti, which was first reported in 2017 as a novel genus and had been isolated from a scalp pustule of a 29-year-old man in Nice, France (1).

Strain SGAir0207 was isolated from an air sample collected in Singapore (global positioning system coordinates 1.350N, 103.689E) using an Andersen-type air sampler and directly impacted onto M9 minimal salts (MP Biomedicals, USA) with carboxymethylcellulose sodium salt (10 g/liter). Isolation of colonies was then carried out by culturing on Trypticase soy agar (Becton, Dickinson, USA) at 30°C. A single colony was then inoculated in lysogeny broth medium (Becton, Dickinson) and incubated overnight at 30°C prior to DNA extraction. Genomic DNA was purified using the Wizard genomic DNA purification kit (Promega, USA) according to the manufacturer’s protocol. A whole-genome shotgun library was constructed with the TruSeq Nano DNA library preparation kit (Illumina, USA) and sequenced on an Illumina MiSeq platform using 300-bp paired-end reads. The same genomic DNA extract was also subjected to long-insert library preparation with the SMRTbell template prep kit 1.0 (Pacific Biosciences, USA), followed by single-molecule real-time (SMRT) sequencing on the PacBio RS II platform.

All the following software programs were used with default settings, unless otherwise stated. Quality control of reads was performed using PreAssembler filter v1 from Hierarchical Genome Assembly Process v3 (HGAP3) (2) for PacBio reads and Cutadapt v1.8.1 (3) for MiSeq reads. A total of 43,999 PacBio subreads (mean length, 13,051 bp; raw read *N*_50_ value, 18,645 bp) were used for *de novo* assembly with HGAP3 implemented in the PacBio SMRT Analysis 2.3.0 package. The draft assembly was polished using the software Quiver (2), and Pilon v1.16 (4) was used for error correcting (–tracks –changes –vcf –fix all –mindepth 0.1 –mingap 10 –minmq 30 –minqual 20 –K 47) using 933,783 MiSeq paired-end reads. The consensus assembly generated 8 contigs (Table 1). Quality Assessment Tool for Genome Assemblies (QUAST) (5) was used to obtain the contig lengths and mean GC content.

**TABLE 1:** Contigs, lengths, coverages, mean G+C content, and accession numbers of SGAir0207

Contig name	Length (bp)	Coverage (fold)	Mean GC content (%)	Accession no.
Chromosome	3,928,779	101	60.80	CP028035
pSGAir0207_1	305,149	114	59.45	CP028036
pSGAir0207_2	389,926	107	60.08	CP028037
pSGAir0207_3	119,784	104	50.29	CP028038
pSGAir0207_4	101,764	94.9	52.72	CP028039
pSGAir0207_5	73,038	50.7	56.67	CP028040
unnamed_6	74,494	41.8	53.48	CP028041
unnamed_7	37,502	39.4	56.05	CP028042

Average nucleotide identity (ANI) was estimated using the software Microbial Species Identifier (MiSI) v1.0 (6), resulting in 84.0% marker similarity and an alignment fraction (AF) of 36% with *N. archeti.* Subsequent BLASTn analysis (BLASTN 2.9.0+) (7) against the NCBI 16S ribosomal RNA (bacteria and archaea) database was performed using the 16S rRNA gene and resulted in 99% identity to *Nissabacter archeti* strain 2134, with 95% query coverage. Together, these results suggest that this isolate is a member of the genus *Nissabacter* but possibly a new species.

The genome annotation using the NCBI’s Prokaryotic Genome Annotation Pipeline (PGAP) v4.2 (8) predicted a total of 4,594 genes, with 4,403 protein-coding genes, 22 rRNA subunits (8 genes for 5S and 7 genes each for 16S and 23S rRNA subunits), 84 tRNAs, 7 noncoding RNAs, and 78 pseudogenes. Functional annotation using Rapid Annotations using Subsystems Technology (RAST v2.0, default parameters with added automatically fix errors, fix frameshifts, backfill gaps, and annotation scheme “Classic RAST”) (9–11) showed that 93 genes were associated with virulence, disease, and defense, with 73 genes potentially linked to resistance to antibiotics and toxic compounds, which may confer infectious competence to this organism. Fifty-two genes from the subsystem were predicted to belong to phages and prophages that were probably integrated into the bacterial genome.

### Data availability.

The complete genome sequences of *Nissabacter* sp. strain SGAir0207 and its seven plasmids have been deposited in DDBJ/EMBL/GenBank under accession numbers CP028035, CP028036, CP028037, CP028038, CP028039, CP028040, CP028041, and CP028042. The SRA accession numbers are SRR8894410 and SRR8894411.
